# Head-mounted digital metamorphopsia suppression as a countermeasure for macular-related visual distortions for prolonged spaceflight missions and terrestrial health

**DOI:** 10.1017/wtc.2022.21

**Published:** 2022-10-12

**Authors:** Joshua Ong, Nasif Zaman, Ethan Waisberg, Sharif Amit Kamran, Andrew G. Lee, Alireza Tavakkoli

**Affiliations:** 1 School of Medicine, University of Pittsburgh, Pittsburgh, PA, USA; 2 Human-Machine Perception Laboratory, Department of Computer Science and Engineering, University of Nevada, Reno, NV, USA; 3 School of Medicine, University College Dublin, Dublin, Ireland; 4 Center for Space Medicine, Baylor College of Medicine, Houston, TX, USA; 5 Department of Ophthalmology, Blanton Eye Institute, Houston Methodist Hospital, Houston, TX, USA; 6 The Houston Methodist Research Institute, Houston Methodist Hospital, Houston, TX, USA; 7 Departments of Ophthalmology, Neurology, and Neurosurgery, Weill Cornell Medicine, New York, NY, USA; 8 Department of Ophthalmology, University of Texas Medical Branch, Galveston, TX, USA; 9 University of Texas MD Anderson Cancer Center, Houston, TX, USA; 10 Department of Ophthalmology, Texas A&M College of Medicine, College Station, TX, USA; 11 Department of Ophthalmology, The University of Iowa Hospitals and Clinics, Iowa City, IA, USA

**Keywords:** design, optimization, performance augmentation

## Abstract

During long-duration spaceflight, astronauts are exposed to various risks including spaceflight-associated neuro-ocular syndrome, which serves as a risk to astronaut vision and a potential physiological barrier to future spaceflight. When considering exploration missions that may expose astronauts to longer periods of microgravity, radiation exposure, and natural aging processes during spaceflight, more severe changes to functional vision may occur. The macula plays a critical role in central vision and disruptions to this key area in the eye may compromise functional vision and mission performance. In this article, we describe the development of a countermeasure technique to digitally suppress monocular central visual distortion with head-mounted display technology. We report early validation studies with this noninvasive countermeasure in individuals with simulated metamorphopsia. When worn by these individuals, this emerging wearable countermeasure technology has demonstrated a suppression of monocular visual distortion. We describe the considerations and further directions of this head-mounted technology for both astronauts and aging individuals on Earth.

## 1. Introduction

**Figure 1.**
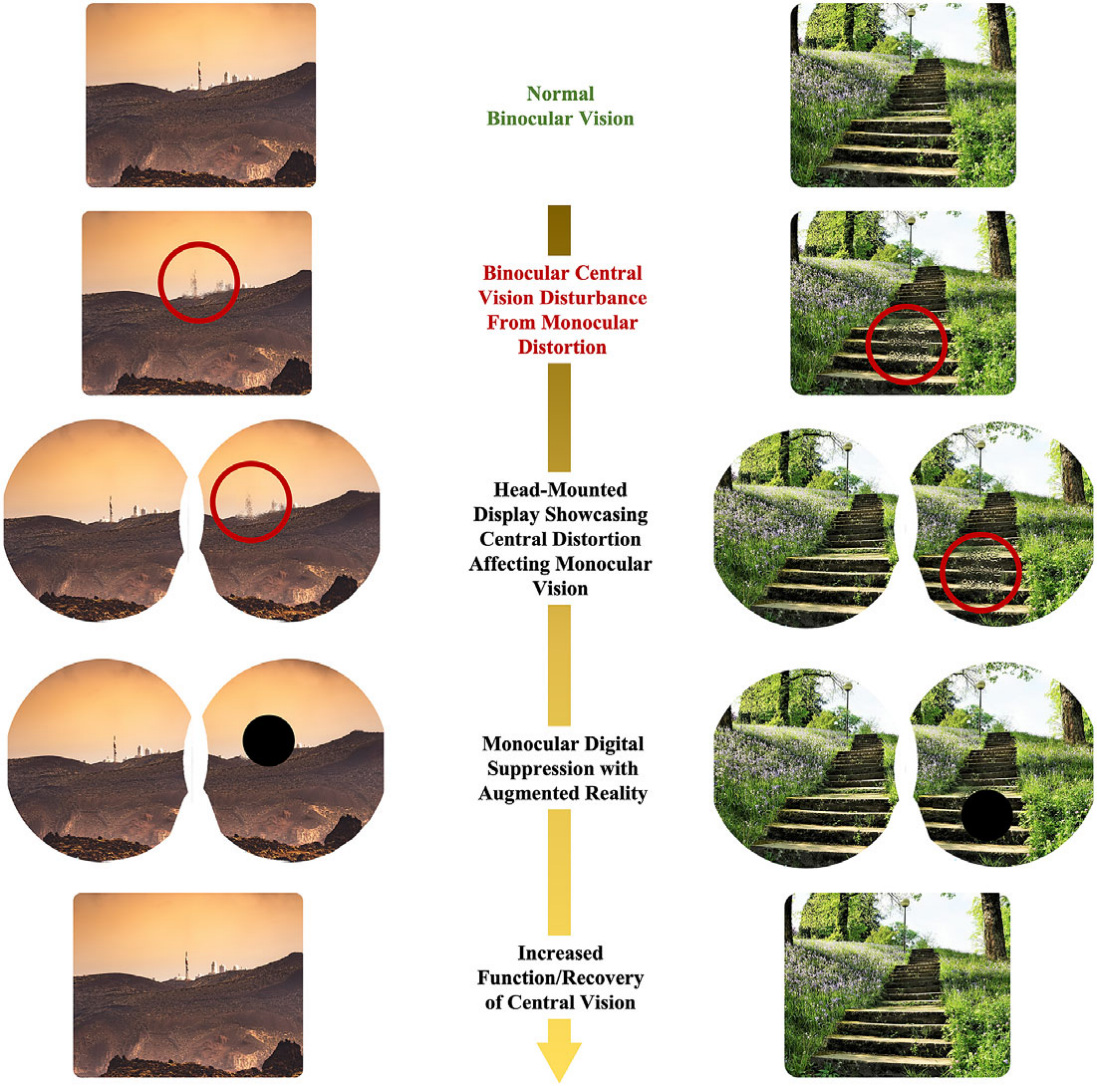
Illustration of digital, monocular suppression on monocular central distortion with head-mounted augmented reality.

**Figure 2.**
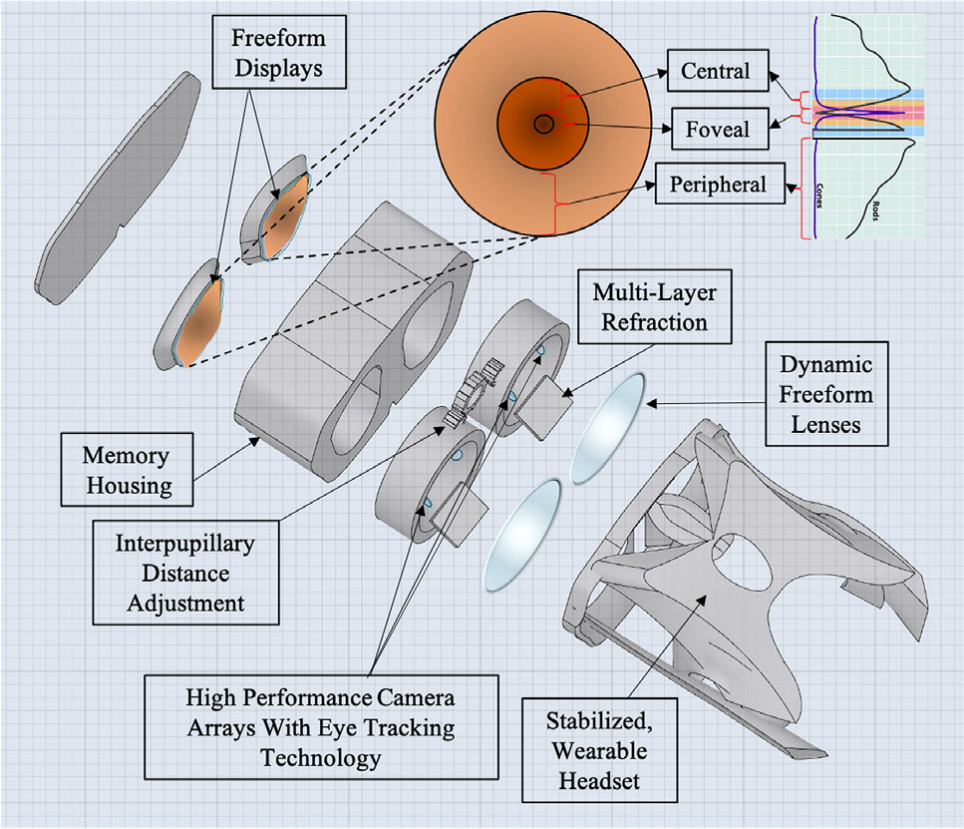
Computer-aided design of head-mounted, comprehensive visual assessment system for spaceflight with foveated rendering.

When exposed to prolonged periods of microgravity, the human body undergoes various physiological changes including muscle atrophy, neuro-ophthalmic changes, and bone density loss (Demontis et al., 2017; Lee et al., 2020). Astronauts are also exposed to high levels of radiation from galactic cosmic rays and solar particle events, increasing various health risks to the human body (Patel, 2020); Davis et al., 2021. Among other factors such as prolonged isolation and a hypercapnic environment, spaceflight harbors a challenging environment for human physiology and health (Patel et al., 2020; McGregor et al., 2021). However, upholding an astronaut’s longitudinal health is critical for mission performance and success (Hackney et al., 2015; Patel et al., 2020). A large consideration for astronaut health is the impact of prolonged spaceflight on vision and ocular structure (Patel et al., 2020; Ong et al., 2022a). Head-mounted, wearable technology may serve as a solution to address several of these considerations. In this manuscript, we discuss potential risks to the macula during deep space exploration such as spaceflight-associated neuro-ocular syndrome (SANS), radiation, and aging. We also discuss common causes of macular disruptions and subsequent irreversible vision loss on Earth. We report the technical development and results of validation studies of head-mounted, digital suppression as countermeasures for macular-related visual distortion (Figure 1). These countermeasures serve to uphold mission performance and are a component in the comprehensive head-mounted visual assessment system being developed to monitor ocular health during spaceflight (Figure 2) (Ong et al., 2022b). In addition, this technology may potentially be applied to individuals on Earth with monocular macular disruptions from various causes such as aging.

## 2. Risk of ophthalmic changes during spaceflight

During the early Shuttle Missions, astronauts began to report vision changes that led to decreased visual performance during spaceflight (Bloomberg et al., 2016). These anecdotal changes led to a progressive, large-scale investigation by the National Aeronautics and Space Administration (NASA) to document functional and structural ocular changes that occur during spaceflight. In 2011, Mader et al. (2011) published the initial report of the neuro-ophthalmic findings observed in a cohort of astronauts after long-duration spaceflight (LDSF). This report found optic disk edema, hyperopic refractive error shifts, choroidal folds, and posterior globe flattening in astronauts after LDSF (Mader et al., 2011; Stenger et al., 2017). The constellation of these neuro-ophthalmic symptoms is now known as SANS. SANS has been assigned an elevated “Likelihood and Consequence” by NASA for Mars missions (Patel et al., 2020). This risk indicates the top priority for the development of countermeasures to mitigate SANS as optic disk edema and choroidal folds present with long-term risks to astronaut vision (Schirmer and Hedges, 2007; Patel et al., 2020; Agrawal and Tripathy, 2021). The underlying pathogenesis behind SANS is not well understood and diverse research avenues have been established to further understand SANS pathophysiology and mitigate the effects of SANS (Laurie et al., 2019; Scott et al., 2019; Ashari and Hargens, 2020; Harris et al., 2020; Kermorgant et al., 2021; Ong et al., 2021).

The macula is a small area of the retina that plays a critical role in central vision and color detection (Medical Advisory Secretariat 2009). Disruptions to this important ocular area can cause severe visual impairment with subsequent inability to conduct daily activities of living such as driving, recognizing faces, and reading (Mitchell and Bradley, 2006). While central vision loss has not been reported in astronauts, there are several considerations for macular health during future spaceflight including prolonged effects of SANS than what is currently known, natural aging processes, and increased radiation exposure to the macula. Any deterioration in macular health poses a risk for astronaut and mission safety in deep space exploration. Although preventative measures are necessary to reduce underlying risks, countermeasures to uphold visual performance during active macular distortions may be useful during missions with limited medical care and resources.

## 3. Macular disruptions in terrestrial health

Macular disruptions are also common in terrestrial health such as age-related macular degeneration (AMD), central serous chorioretinopathy (CSCR), and diabetic macular edema (DME) (Wang et al., 2008; Lim et al., 2012; Bahrami et al., 2016). AMD is one of the leading causes of irreversible blindness in the elderly population (Lai and Landa, 2015). Risk factors for AMD include aging and smoking (Heesterbeek et al., 2020). A subtype of AMD, neovascular AMD (nAMD), is characterized by choroidal neovascularization (CNV) often leads to rapid and severe central vision loss. If left untreated, it has been reported that roughly 50–60% of nAMD eyes with subfoveal CNV may lose 6+ lines of vision in the course of 2–3 years (Hobbs and Pierce, 2022). Therapeutic advances for nAMD have significantly reduced blindness, although there are still unmet needs for nAMD refractory treatment and patients encountering barriers to receiving treatment (Patel and Sheth, 2021; Varano et al., 2015). These current obstacles may lead to the pathological course of irreversible vision loss in this macular disease.

CSCR is a common retinopathy that can also damage the macula. It is characterized by serous detachment of the neurosensory retina involving the macular region (Semeraro et al., 2019). Patients often experience blurred vision, scotoma in central vision, and metamorphopsia. CSCR is more likely to occur in men, with an annual incidence rate of 10 per 100,000 men (Liew et al., 2013). Although CSCR is often self-limiting and resolves spontaneously within a few months, persistent CSCR can lead to irreversible vision loss due to atrophy of the retinal pigment epithelium (Sartini et al., 2019; Semeraro et al., 2019). DME is one of the leading causes of vision loss in diabetic patients (Musat et al., 2015). It is estimated that over 460 million people have diabetes worldwide which is anticipated to increase by approximately 50% by 2045 (Saeedi et al., 2019). This trend points towards an increased risk of irreversible vision loss in this patient population.

Central vision loss from macular disorders can result in a loss of independence and quality of life. Elderly individuals affected by AMD often experience loss of independence (e.g., no longer able to drive) and the full ability to enjoy retirement activities which can be highly distressing (Gehrs et al., 2006). Madheswaran et al. (2021) reported in a qualitative study that AMD has a severe negative impact on the quality of life, often due to the inability to recognize faces and feeling social disconnect. As such, there is a direct need for accessible solutions to help rehabilitate and reduce the effects of vision loss from macular disruptions. Given its accessibility and portability components, wearable head-mounted technology has emerged as a promising innovation for terrestrial ophthalmic disorders including AMD, glaucoma, and amblyopia (Mohaghegh et al., 2016; Miao et al., 2020; Stapelfeldt et al., 2021). In the following sections, we detail the development of a head-mounted, digital suppression as a potential countermeasure for macular-related visual distortions. We discuss technical hardware considerations for optimizing the deployment of this wearable technology countermeasure including precision eye-tracking and high resolution for visual fidelity. We also report early validation study results with this noninvasive countermeasure with simulated metamorphopsia.

## 4. Materials and methods

In this section, we first discuss the materials and methods to simulate metamorphopsia utilizing virtual reality/augmented reality (VR/AR). We then report the development of dark spot digital suppression and the procedure utilized for individuals with simulated metamorphopsia. The study was conducted under the University of Nevada, Reno Institutional Review Board (IRB) Study ID Number 1784519–1 titled: Comprehensive Assessment, Simulation and Management of Visual Impairment.

## 5. Headset hardware

**Figure 3.**
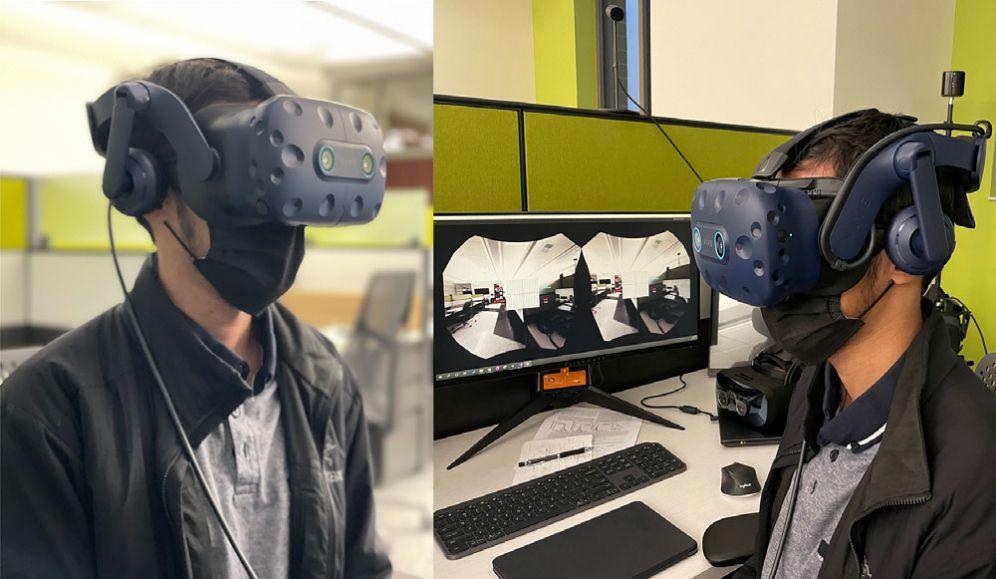
Hardware for the digital metamorphopsia suppression countermeasure. HTC Vive Pro Eye headset with user undergoing augmented reality with overlaying Amsler Grid.

To simulate metamorphopsia, the HTC Vive Pro Eye headset was used as seen in Figure 3. Several technical aspects were taken into consideration when selecting a head-mounted technology to run the countermeasure software for this early validation study. The HTC Vive Pro Eye allows for precision eye-tracking, which allows for additional analytics when conducting assessments. This data will be particularly helpful during spaceflight assessments where there is a higher risk for vestibulo-ocular changes and focus impairments (Bloomberg et al., 2016). In addition to assessments, gaze-tracking allows for additional insight on what the user is viewing through the system the most which may allow for optimal performance-based feedback. The headset has a field of view of 110° (diagonal), dual OLED displays with a combined resolution of 2,880 *×* 1,600 pixels, and a pixel density of 615 pixels per inch per eye. With currently available head-mounted technology, these specifications represent professional-grade levels in extended reality hardware. Achieving the highest visual fidelity is of utmost importance for this technology for optimal astronaut performance and terrestrial health benefits for individuals with irreversible central vision loss. The headset is relatively light, weighing at 6.05 lbs. which allows for users to wear the technology for extended periods of time without fatigue. The technology also optimizes GPU workload with foveated rendering. This technology allows for the optimization of graphic fidelity in the wearer’s central vision and lowers the resolution in the periphery. This technology is particularly helpful when seeking to optimize an astronaut’s central visual performance. In resource-limited areas, such as spaceflight, these aspects of efficient computing are highly valuable.

The participants are helped to wear the headset properly and adjustments are made to ensure a comfortable fit. The lens distance and interpupillary distance can also be modified while the participant is in the VR environment to ensure the device is fully calibrated. The results of the user study were recorded on a CSV file. An Unreal Engine Plugin (SRanipal) was used to track the gaze of the participants and dynamically suppress the distortions on the affected eye.

## 6. Metamorphopsia simulation design

**Figure 4.**
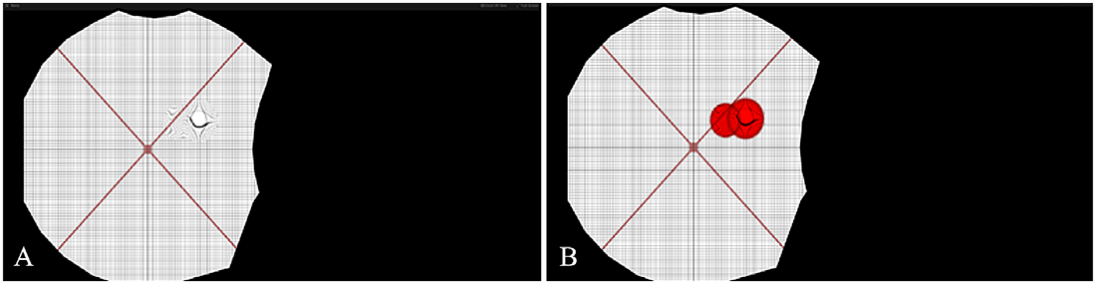
Simulation of metamorphopsia with the perceptual deficit model (PDM). (a) showcases a simple metamorphopsia created with a single Gaussian kernel and (b) showcases a more complex metamorphopsia with multiple (denoted by the two red circles) Gaussian kernels applied in conjunction.

Metamorphopsia is a very common form of visual distortion in various terrestrial macular disorders (e.g., AMD). Metamorphopsia is often described as a distortion or deviation of straight lines or instability of vision (Midena and Vujosevic, 2015). In an assessment of metamorphopsia, approximately 45% of patients with AMD perceived some level of optical distortion (Xu et al., 2018). If astronauts encounter these distortions during spaceflight, mission-critical tasks could be compromised. Therefore, it is important to identify perceptual distortions in individuals and construct appropriate countermeasures. However, the considerable variation in distortion shape, size, and location creates challenges for accurate assessment and countermeasure. Our proposed methodology considers and builds upon a physical-based metamorphopsia modeling called Perceptual Deficit Modeling (PDM) (Zaman et al., 2020). In this model, a mixed reality system simulates distortions in one of the normal healthy eyes and has the participant reconstruct the perceived distortion in their other eye (Figure 4) (Zaman et al., 2020). However, this approach requires extensive cooperation from the participant and is a cause of considerable cognitive stress. As most AMD patients are elderly, a more straightforward process is desired. In this section, we summarize the PDM approach and describe how we improved the modeling and rehabilitation steps.

PDM describes perceptual deficit or metamorphopsia in AMD patients (Table 1) with four different parameters: localization, size, shape, and luminance perception:
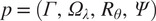
where 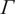 is the luminance sensitivity loss, 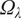 is the covered area, 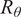 and 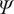 are the rotational and spatial distortion of the visual field. The model utilizes multiple instances of circular metamorphopsia to create a more complex shape (Figure 4) through the Gaussian Mixture Model (GMM). The parameter 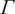 was used in conjunction with 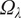 in the following way:
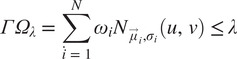
where 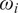 denotes the luminance loss, 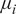 denotes the center and 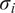 is the standard deviation of the Gaussian kernels, while 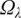, limits the boundary of the kernels to 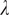. 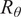 is a rotational operation that rotates the kernels tangentially in proportion to the distance to the kernel center. Finally, 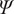 is a spatial distortion operation that stretches or squeezes the kernel radially. This approach unnecessarily complicates the process of metamorphopsia parameterization by connecting all the parameters to the same Gaussian kernels. We separated the kernels of perceptual loss, rotational distortion, and spatial distortion, effectively modeling the same complexity of three independent kernels into one:
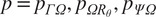

**Table 1.** Parameters designed to replicate the perceptual deficit seen in age-related macular degeneration

Parameters	Range	Step size
Distortion	0 ≤ d ≤ 100	37.2
Sigma	0 ≤ λ ≤ 0.75	0.0075
Mean	0 ≤ (u, v) ≤1	0.1
Weight	0 ≤ w ≤ 1	0.02
Rotation	−360 ≤ θ ≤ 360	2.0

where each kernel only shares size parameters. Additionally, the properties of the underlying Amsler grid are parameterized to improve the monitoring capability of the system by permitting finer grids, variable grid line width, and grid chromaticity. Furthermore, diagonal lines were added to the grid to reinforce the point of fixation, as macular vision loss may make it difficult to find the central fixation point. However, the most fundamental change in the modeling is from the user’s perspective.

**Figure 5.**
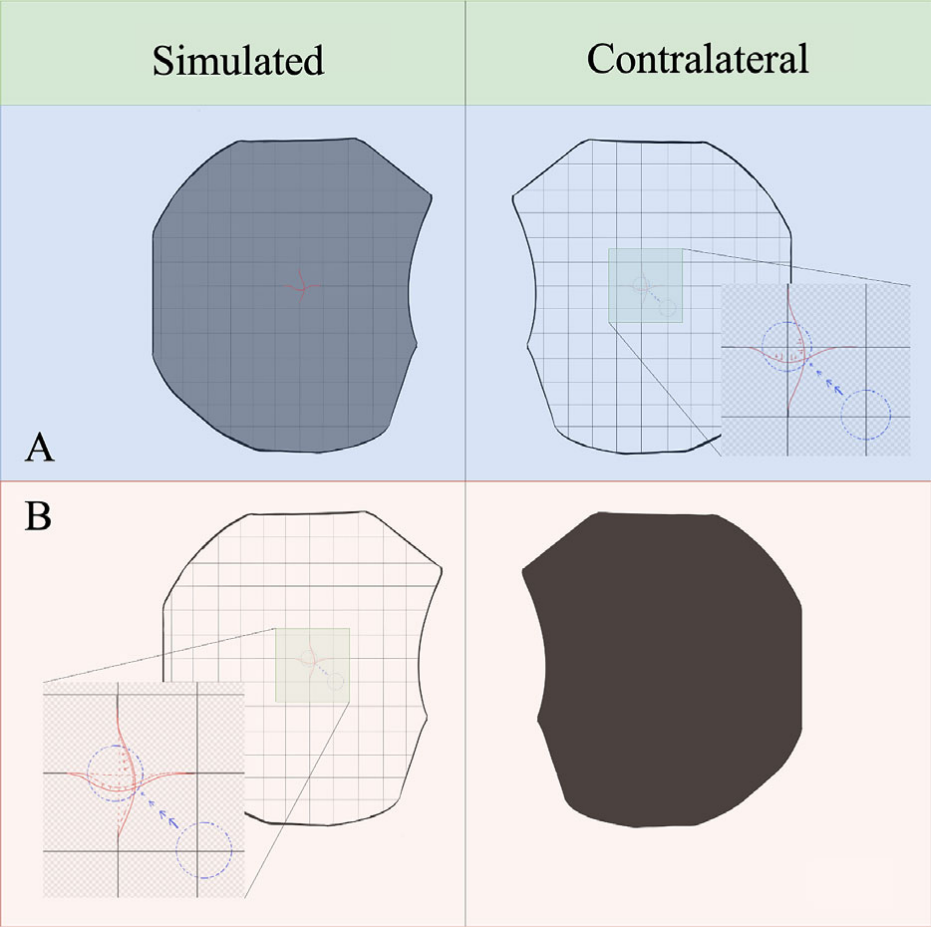
(a) Standard perceptual deficit modeling (PDM) approach with recreation of the metamorphopsia on the contralateral eye. (b) Our new approach to digital metamorphopsia suppression by applying corrective distortion to the affected eye directly. This new approach may also allow for binocular macular distortions as the contralateral eye display is turned off.

In the standard PDM module, the user reconstructs the perception in the impaired eye in their normal eye. For example, in healthy subjects, the approach first simulates a metamorphopsia with given parameters in one of the eyes, 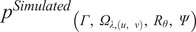. The task of the participant is to create a similar metamorphopsia in their contralateral eye. Initially, a PDM is overlaid on the contralateral eye with parameter values 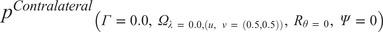. The participant can change the parameters to find the ideal case,
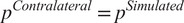


This approach is similar to the traditional article-based Amsler grid test where the participant attempts to draw the perceptual distortion that they are experiencing with a pen. To avoid binocular interaction when trying to reconstruct the metamorphopsia in the other eye, the display input to the test eye is turned off. That display is later turned back on when the subject wants to recheck. This approach (Figure 5a) is challenging because the position, size, and shape information may be lost during ocular shift caused by the monocular display. Furthermore, due to the subjective bias to the reconstructive parameters, the corrective distortion to counter the perceptual deficit caused by the simulated distortion is not guaranteed when a reverse of the 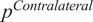
is applied.

To address such limitations, we directly model the corrective action in the impacted eye. For that purpose, a default metamorphopsia 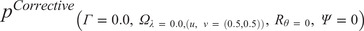
is drawn on the affected eye itself, instead of the contralateral eye. The participant then manipulates the parameters of this distribution but with a completely different goal compared to the PDM approach. Now, instead of reconstruction, the aim is to make the distortion disappear. This makes it possible to precisely identify the metamorphopsia characteristics. An added benefit of this approach is that we can now model bilateral macular distortions with corrective distributions for each eye separately. This is possible because the contralateral eye display is always turned off during corrective assessment of the test eye (Figure 5b).

**Figure 6.**
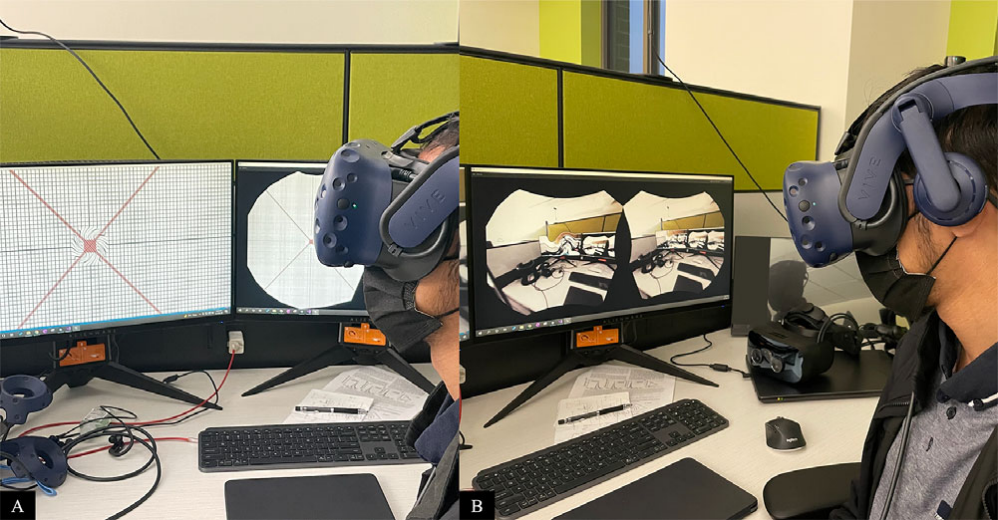
Simulated metamorphopsia on an Amsler Grid (a) and through the augmented reality camera feed (b).

Furthermore, simply applying the corrective distortion to the see-through camera feed appears to improve vision in some patients. By applying the suppression through a camera feed with an AR system, individuals with monocular macular disruptions may be able to complete daily tasks more effectively despite experiencing functional unilateral macular decline (Figure 6).

## 7. Dark spot digital suppression design

**Figure 7.**
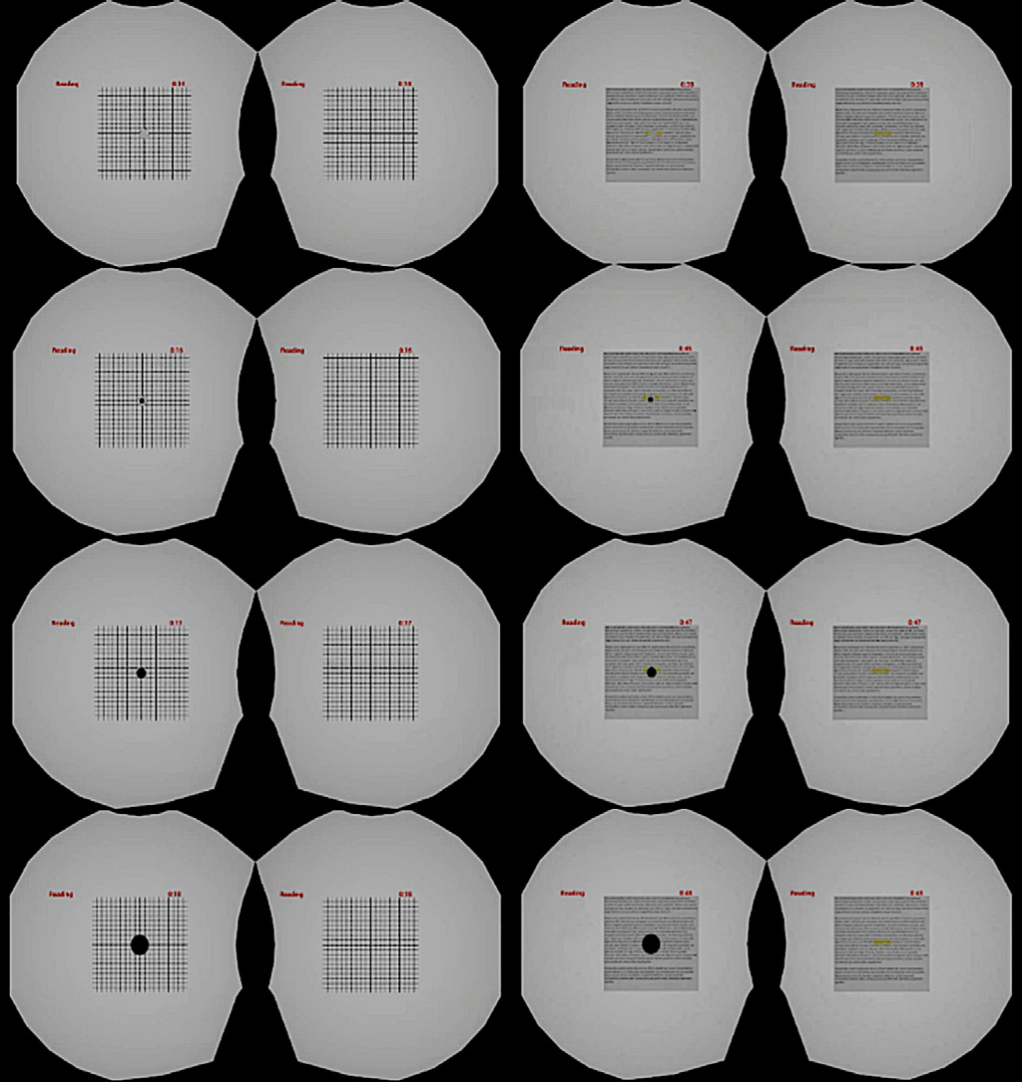
Dark spot suppression of various sizes in both Amsler grid (left) and reading tasks (right).

As the participants directly model the corrective distortion of their metamorphopsia, very little additional suppression is required. A dark circular spot overlays the area of metamorphopsia in the VR/AR device for the affected eye. An important consideration for this for creating a digital suppression is to only block out the distorted part and not exceed its boundary so as not to cause additional visual information loss. However, if the suppression is too small, the peripheral distortion may cause additional perceptual loss. In our validation studies, we tested various sizes of digital suppression relative to the size of the distortion, including 25 and 120% (Figure 7).

## 8. Simulated metamorphopsia with dark spot digital suppression procedure

Once the participant is wearing the headset, both eyes are presented with a standard Amsler grid. A distortion questionnaire is used to establish control measurements. A random metamorphopsia template is then simulated in one of the eyes. Suppressing dark circles were superimposed on the simulated metamorphopsia at 0, 25, 90, and 120% relative to the size of the distortions. These suppression sizes are based on preliminary experiments that test different countermeasure tradeoffs. For example, if 25% suppression is effective, it means that most of the metamorphopsia distribution is concentrated in the central area and the outer area is only slightly noticeable. Ninety percent and 120% suppressions were chosen because of the property of Gaussian distributions which was used in the distortion modeling. These distributions lack a clear boundary and 90% of parameter λ is perceptually close to 100% but allows the user to see slightly more of the field of view. One hundred and twenty percent is selected because it always covers and noticeably exceeds any distortion. Future studies will likely explore different parameters of dark spot sizes for fine-tuning of suppression. Subjects then responded to questions regarding the ability to perceive the distortion after levels of suppression via Amsler grid and text-based evaluations. The participant then answers questions related to their perception. The perception was rated between 0 and 5, where 0 signified imperceptible distortion and 5 signified the highest distortion. In the reading task, the participants reported how easily they were able to read some highlighted texts. The text was composed of random Latin so that participants could not simply guess the highlighted word from context. Healthy participants had no trouble reading the highlighted text when no distortion was introduced. To determine how difficult reading became once a simulated metamorphopsia was induced in one eye, we used a subjective scoring method. Five signified complete illegibility and 0 signified complete legibility.

The experiment uses a repeated measures design in which the affected area in the visual field is then increasingly suppressed in a stepwise manner. At each increasing level of suppression, the participant will answer questions regarding their perception of the simulated distortion. The first suppression area at which the distortion cannot be perceived is recorded for each eye, and when the suppression is first noticed is also recorded. The independent variables used in the experiment are the six different conditions associated with different levels of simulated metamorphopsia. It is worth noting that when the eyes of the subject move, the suppression in the simulation moves as well. This allows the healthy eye to fill in the distorted area, and replace distortions created by the scotoma. The data consists of questionnaires with six discrete outcomes does not meet the requirements for analysis of variance (ANOVA). Therefore, nonparametric analysis is performed to evaluate the performance. To perform the statistical analysis, we assigned a numerical value to each of the categories of the dependent variables from the range of 0 to 5, that is, imperceptible distortions category was assigned a value of 0 while the highest distortion category was given a value of 5. We used Chi-square analysis for evaluating the results.

## 9. Participants and methods

Eighteen healthy subjects (normal eyes with no history of ocular pathology) were recruited for this validation study. Six females and 12 males with an average age of 28.59 ± 3.22 years were included. Individuals with any history of prior ocular disease were excluded. All participants underwent simulated metamorphopsia with the head-mounted VR system mentioned in the materials section.

## 10. Results

All individuals responded that the headset was comfortable, and that they could read the information on the screen clearly.

**Figure 8.**
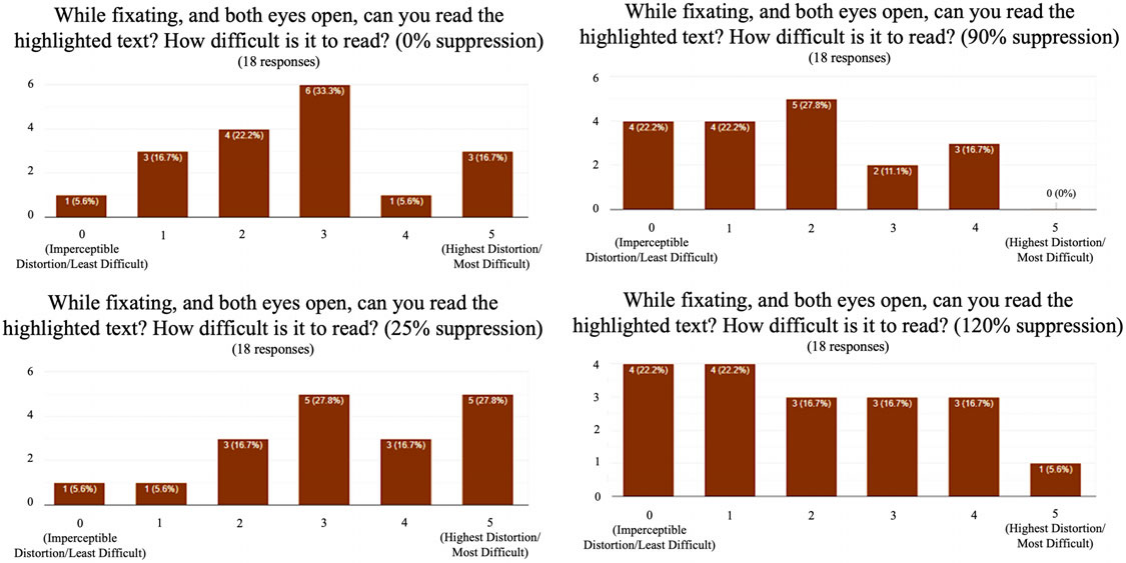
Graphical results from highlighted legibility reading task with digital suppression. Ninety percent suppression revealed the highest mean legibility.

Chi-square analysis shows that using 25% dark spot suppression of the distortion reduced the mean perception of distortion to four (*p* = .005). Ninety percent of dark spot suppression of the distortion reduced the mean perception of distortion to one (*p* < .005). One hundred and twenty percent dark spot suppression eliminated all perception of the distorted grid with a distortion of 0 (*p* = <.005). The highest mean legibility during the reading task was found at 90% suppression with a score of 1.78 (Figure 8). The mean perception of distortion was more reduced in the reading task (0.17) compared to the Amsler grid task (0.61). No adverse events were experienced throughout this noninvasive protocol.

## 11. Discussion

This early validation study demonstrates that this digital suppression technique in individuals undergoing simulated unilateral metamorphopsia may reduce central vision distortion in binocular vision. There was a statistically significant binocular compensation with 90 and 120% VR-based suppression of simulated metamorphopsia. Interestingly, dark spot suppression at 120% was effective in removing the distortion but also introduced another impairment because the dark spot was more noticeable, thus indicating that 90% suppression may be the ideal size for suppression. Fine-tuning the suppression in terms of dark spot size may help to further improve legibility. As these early pilot studies for this emerging technology/technique contain small sample sizes, larger studies are being planned to further understand the effects of this digital countermeasure, particularly in conducting real-life tasks that mimic daily activities of living utilizing AR. As the hardware of wearable technology systems continues to advance in visual fidelity, the results from this countermeasure may be further improved. Limitations in this early validation study include metamorphopsia simulation only, limited size choices in dark spot suppression, and a small sample size. It is anticipated that these limitations are addressed in future studies.

**Figure 9.**
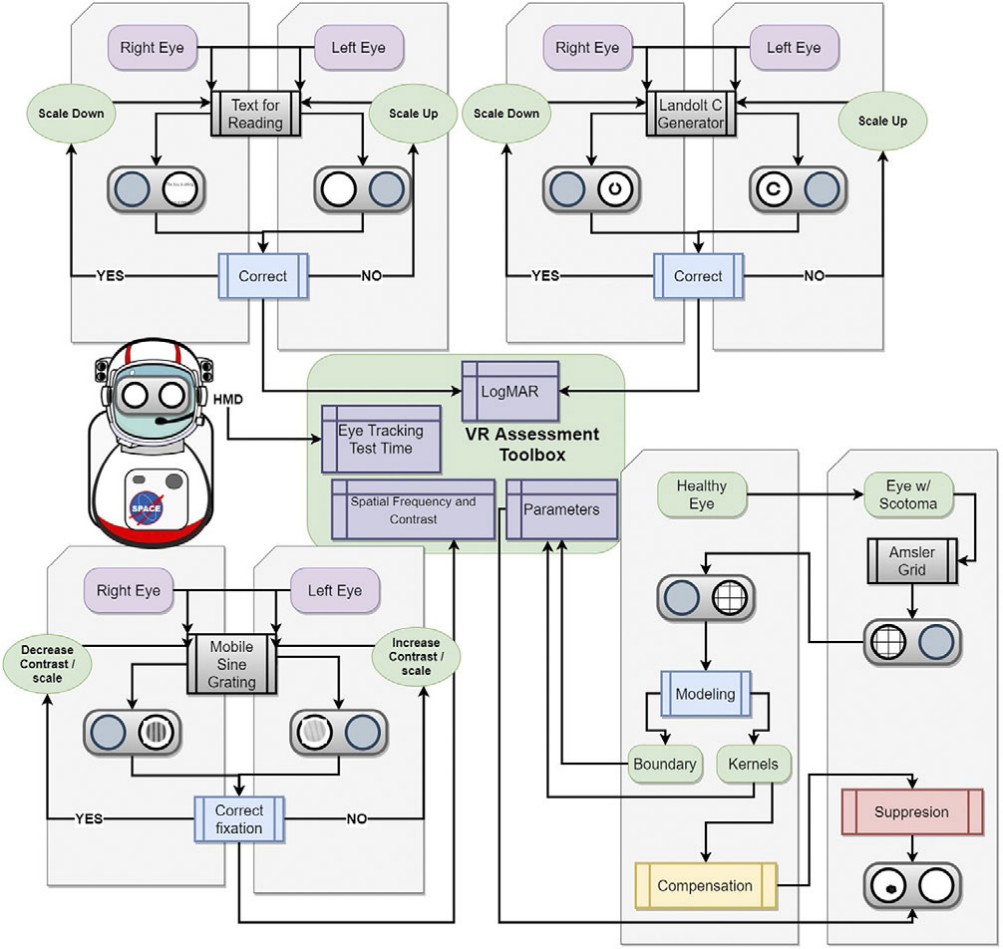
Roadmap to a comprehensive mixed reality head-mounted display to monitor functional vision and provide digital suppression for astronauts who undergo long-duration spaceflight. The toolbox includes several assessments including reading visual acuity, far visual acuity, contrast sensitivity, and Amsler grid. The toolbox aims to collect extensive metadata including eye-tracking, performance against a particular stimulus, stimuli response time, and session time which can be combined with more conventional data such as logMAR visual acuity and contrast sensitivity function to reduce the impact of these subjective tests and increase their predictive power.

This digital suppression technique is one aspect of the development of a comprehensive multi-modal, head-mounted display to monitor functional vision in astronauts (Figure 9) (Ong et al., 2022b). As astronauts are exposed to longer periods of microgravity and increased radiation, this countermeasure seeks to address the potential vision loss that may arise from these increased risks. These potential risks to the macula include worsening SANS, radiation exposure, and macular changes from natural aging.

Future planetary missions will likely expose astronaut travelers to microgravity longer than what is currently known, thus, SANS may affect ocular structures more so than what is currently documented. Current SANS documentation includes choroidal folds in the papillomacular bundle area (Stenger et al., 2017). Terrestrial reports of chronic choroidal folds have been observed to result in alterations of the macula resulting in choroidal fold-related maculopathy and deterioration of central vision (Olsen et al., 2014; Grosso et al., 2020). During prolonged planetary missions with years or even a lifetime in space, it is expected that astronauts will undergo natural aging processes; this process may also be affected by the unique stressors of spaceflight such as increased radiation exposure. In addition, as spaceflight seeks to expand to the civilian population (Stepanek et al., 2019), anticipation of various macular diseases that occur in the general population may be helpful for future spaceflight. AMD is a common macular disease that is estimated to account for 9% of blindness worldwide (Stahl, 2020). NASA has monitored for AMD in astronauts through the Vision and Aging in the Astronaut Population study which did identify AMD in the astronaut population, although at a lower prevalence compared to the general population of age 50 and older (NASA, 2003, 2010). Another consideration during deep space exploration includes the effects of increased radiation exposure on the human body, including the macula. Beyond the Earth’s magnetosphere, astronauts are exposed to space radiation of high-charge high-energy particles including solar particle events (SPE) and galactic cosmic rays (GCR). GCRs arrive at a steady rate and pose a serious concern for LDSF such as missions to Mars (Badhwar and O’Neill, 1994; Chancellor et al., 2014; Patel, 2020). Terrestrial radiation maculopathy is an occlusive microangiopathy of the retina, which can impair visual acuity and lead to irreversible blindness (Guyer et al., 1992). Out of the few astronauts that traveled outside of the Earth’s geomagnetosphere, many experienced light flashes after their eyes were dark adapted. This was believed to be caused by cosmic ray nuclei striking the retina (Pinsky et al., 1974). These light flashes were generally white and colorless and were described as either a spot, a flash, or a cloud-like shape. The rate of these events were recorded, and this rate was compatible with the hypothesis that cosmic ray nuclei were causing the light flashes (Pinsky et al., 1974). Although the exact mechanism of how these cosmic rays produce light flashes is unknown, it is believed to be either from an interaction of ionizing radiation with the retina or through the Cherenkov effect. Particles carrying an electric charge traveling through a transparent medium faster than the phase velocity within that medium and causes a brief flash of light (i.e., a Cherenkov effect). Since dark adaptation only involves localized changes to retinal tissue, this supports the theory of ionizing radiation directly interacting with the retina (NASA, 1971; Osborne et al., 1975).

This digital suppression technique may have strong applications for optimizing the quality of life and vision on Earth. There are various age-related risks to the macula, including AMD which is one of the leading causes of irreversible blindness for aging individuals on Earth over 50 (Birch and Liang, 2007). Neovascular AMD has been shown to severely impair quality of life in various studies (Slakter and Stur, 2005). Central visual field loss can impair an individual’s ability to do daily terrestrial tasks such as reading, recognizing friends and family, and driving (Stahl, 2020). Aligned with losing independence, depression has been found to more prevalent in patients with AMD compared to the general elderly population (Gehrs et al., 2006; Casten and Rovner, 2008). In terms of monocular blindness from advanced AMD, individuals with blindness in one eye were found to be significantly more distressed than those who were blind in both eyes (Williams et al., 1998). Additional ophthalmic diseases that also affect the macula include CSCR and DME (Nicholson et al., 2013; Browning et al., 2018). Both macular diseases have also been found to reduce the quality of life (Gonder et al., 2014; Sahin et al., 2014). Although advances in treatment have been found to reduce blindness in many macular diseases, there is still a need to help address the effects the irreversible vision loss. Thus, this head-mounted monocular digital suppression technique may be able to help alleviate distress and increase the quality of life for those suffering from monocular macular disruptions.

As visual impairment can severely impact the quality of life, terrestrial approaches to enhance vision with various wearable technologies have been explored. Space-based research played an integral part in the historical pioneering of head-mounted vision enhancement technology. The Low Vision Enhancement System (LVES) was a novel technology for head-mounted low vision enhancement technology in the 1990s, developed in conjunction with NASA, utilizing technology in computer processing of satellite images and head-mounted vision enhancement technology intended for the space station (Massof and Rickman, 1992; NASA, 1995). Other head-mounted technologies have also been developed throughout the years and have promising applications for low vision enhancement (Deemer et al., 2018). A head-mounted vision enhancement device known as eSight has been shown to immediately improve facial recognition, reading ability, and activities of daily living for visually impaired participants in a multicenter study. This device allows individuals to manually adjust contrast and magnification to aid in performing daily living tasks (Wittich et al., 2018). Several research groups have also proposed external, wearable camera technology with implantable retinal prostheses as a potential countermeasure for retinal degeneration (Tsai et al., 2009; Maghami et al., 2014). Tsai et al. developed a system where a wearable camera is worn by an individual that sends images to a portable processor, which then transmits to an intra-ocularly implanted device with subsequent activation of microelectrodes (Tsai et al., 2009). While the head-mounted technology described in this manuscript is being developed for spaceflight, there is a high potential to apply this technology for individuals on Earth that also have unilateral central vision loss.

## 12. Conclusion and future work

This wearable, head-mounted technology that showcased digital suppression of monocular central visual distortions in these early validation studies continues to undergo iterations of refinement to further support mission performance. In addition, this technology may be applied terrestrially to individuals that experience unilateral metamorphopsia (e.g., macular hole) in the future. Further studies with AR must be conducted to further understand the effect of this countermeasure on mission-related tasks and daily activities of living.

Current studies with this noninvasive technology are also being tested in patients with unilateral macular holes. An additional technique for individuals with a macular hole has also been developed; individuals with a unilateral macular hole first wear the headset and both eyes are presented with a standard Amsler grid. The individual is then asked to fixate to the point where the two diagonals intersect. Secondly, they are asked whether they notice any distortions. Next, they identify the center of the metamorphopsia by telling the examiner to move a small black dot in some specific direction. The examiner then increases or decreases the size of a red circle until it completely covers the area of the distorted macula. The examiner then rotates the covered distortion tangentially in the clockwise direction. If the participant reports that the distortions are getting worse, the examiner rotates in the opposite direction. If the distortion is still not completely imperceptible, the examiner then radially contorts the metamorphopsia inward. If inward contortion exhibits worse results, outward stretching is attempted. Once the corrective distortion is recorded, a see-through AR mode is enabled. Early preliminary results with a small sample size of individuals with a unilateral macular hole have shown that after being corrected with our unilateral metamorphopsia modeling approach, all individuals stated that they were unable to perceive any distortion in the macular region. These encouraging preliminary results with an additional technique suggest the possibility of combining these two techniques for future studies.
